# *Siphoderina hustoni* n. sp. (Platyhelminthes: Trematoda: Cryptogonimidae) from the Maori snapper *Lutjanus rivulatus* (Cuvier) on the Great Barrier Reef

**DOI:** 10.1007/s11230-022-10031-x

**Published:** 2022-05-12

**Authors:** Storm B. Martin, Scott C. Cutmore

**Affiliations:** 1 Centre for Sustainable Aquatic Ecosystems, Harry Butler Institute, Murdoch University, 90 South Street, Murdoch, WA 6150 Australia; 2 School of Biological Sciences, The University of Queensland, Brisbane, QLD 4072 Australia

## Abstract

A new cryptogonimid trematode, *Siphoderina hustoni* **n. sp.**, is reported, collected off Lizard Island, Queensland, Australia, from the Maori snapper *Lutjanus rivulatus* (Cuvier). The new species is moderately distinctive within the genus. It is larger and more elongate than most other species of *Siphoderina* Manter, 1934, has the shortest forebody of any, a relatively large ventral sucker, a long post-testicular zone, and is perhaps most recognisable for the substantial space in the midbody between the ventral sucker and ovary devoid of uterine coils and vitelline follicles, the former being restricted to largely posterior to the ovary and the latter distributed from the level of the anterior testis to the level of the ovary. In phylogenetic analyses of 28S ribosomal DNA, the new species resolved with the other nine species of *Siphoderina* for which sequence data are available, all of which are from Queensland waters and from lutjanid and haemulid fishes. Molecular barcode data were also generated, for the ITS2 ribosomal DNA and *cox*1 mitochondrial DNA markers. The new species is the first cryptogonimid known from *L*. *rivulatus* and the first metazoan parasite reported from that fish in Australian waters.

## Introduction

The tropical and subtropical waters off the coast of Queensland, Australia are among the best understood worldwide for the trematode fauna exploiting marine bony fishes. In particular, from nowhere else can a similarly broad trematode fauna be considered so well characterised with molecular barcode data (see Cribb et al., 2016; also Bray et al., 2016). Among the body of knowledge accumulated in Queensland in recent decades, select trematode groups have been more comprehensively investigated than others. The Cryptogonomidae Ward, 1917 (Platyhelminthes: Trematoda) is among the larger trematode families well represented in Queensland waters which has been subject to recent intensive study.

Aside from a few freshwater species (Cribb 1985; 1986; Miller & Adlard, 2020), a single species from the temperate waters of the Great Australian Bight (Kurochkin & Korotaeva, 1982), and a few scattered reports of incompletely identified worms (Daddow & Jamieson, 1983; Hooper, 1983; Jamieson & Daddow, 1982), the known cryptogonomid fauna of Australia is from tropical and subtropical marine waters in Queensland, with a few species also reported from tropical waters off Western Australia (Miller & Cribb, 2005; 2007a;b;c; 2008a;b; 2009; 2013; Miller et al., 2009a;b; 2010a;b; 2018). All but one of the marine cryptogonomids reported from Queensland are known only from fishes belonging to the Lutjanidae (snappers and fusiliers) and Haemulidae (sweetlips); the exception is *Mitotrema anthostomatum* Manter, 1963, instead found in epinepheline serranids (groupers) (Cribb et al., 1996; 2001; Lester & Sewell, 1989; Olson et al., 2003).

*Siphoderina* Manter, 1934 is the largest genus within the Cryptogonimidae, currently comprising 47 recognised species, known from a variety of bony fishes in tropical and subtropical waters worldwide. Nine species are known from Australian waters (Miller & Cribb, 2008a). The genus concept is not especially distinctive, rather it is defined for the presence of enlarged oral sucker spines together with a combination of generalised crypotogonimid characters and a lack of specialised characters. The presence of oral sucker spines is essentially the only feature distinguishing the concept of *Siphoderina* from *Metadena* Linton, 1910, another large repository for species of generalised form (Miller & Cribb, 2008b). The size of *Siphoderina* is mostly a consequence of a recent synonymy with *Paracryptogonimus* Yamaguti, 1934, proposed by Miller & Cribb (2008b) due to a lack of morphological distinction between the respective type-species. *Siphoderina* has also absorbed, via synonymy, the smaller concepts of *Lappogonimus* Oshmarin, Mamaev & Parukhin, 1961 (see Miller & Cribb, 2008b) and *Pseudallacanthochasmus* Velasquez, 1961 (see Miller & Cribb, 2008a). Conversely, recent work incorporating molecular based phylogenetic analyses has prompted the proposal of several new genera which have each received species previously recognised in *Siphoderina*, specifically: *Adlardia* Miller, Bray, Goiran, Justine & Cribb, 2009a, *Euryakaina* Miller, Adlard, Bray, Justine & Cribb, 2010a, *Retrovarium* Miller & Cribb, 2010a and *Varialvus* Miller, Bray, Justine & Cribb, 2010b (see Miller & Cribb, 2007a; 2009a; 2010a;b). Here we report a distinctive new species of *Siphoderina* in Queensland waters from a previously unexamined fish.

## Materials and methods

### Host and parasite collection

As part of a general ichthyoparasitological survey of fishes at Lizard Island, Queensland, a single Maori snapper *Lutjanus rivulatus* (Cuvier) was collected *via* spearfishing in November 2016. The body cavity was opened and viscera removed and examined under stereo microscope in saline solution (three parts tap water to one part sea water). The gut (stomach, intestine and pyloric caeca) was opened and trematodes removed from among the villi. Following initial examination, the gut was examined for trematodes using the gut-wash approach described by Cribb & Bray (2010). Trematodes were fixed, without pressure, in near-boiling saline and preserved in 80% ethanol. These standard protocols are described in further detail in Cribb & Bray (2010).

### Morphological study

For morphological study, specimens were rinsed of ethanol in distilled water, stained in Mayer’s haematoxylin, destained in dilute HCl (1%), neutralised in dilute NH_4_OH (1%), dehydrated in ethanol solutions of increasing concentration (50, 70, 90, 95, 100, 100%), cleared in methyl-salicylate, and mounted in Canada balsam. Measurements were made using an Olympus SC50 digital camera mounted on an Olympus BX-53 compound microscope with cellSens Standard imaging software. Measurements are in micrometres (µm) and are expressed as a range, followed by the mean in parentheses; length is followed by width where applicable. The oral sucker spine count was taken from 26 specimens, spine length averaged from 5 spines per specimen, and egg dimensions averaged from 10 eggs per specimen for 8 specimens. Line drawings were made with a drawing tube fitted to the same compound microscope, and digitised with Adobe Illustrator CS6 software. Type-specimens are lodged in the Queensland Museum (QM), Brisbane. To comply with the regulations set out in article 8.5 of the amended 2012 version of the International Code of Zoological Nomenclature (ICZN, 2012), details of the new taxon have been submitted to ZooBank; the Life Science Identifier (LSID) is reported in the taxonomic summary.

### Generation of sequence data

Genetic sequence data were generated for the cytochrome *c* oxidase 1 mitochondrial barcoding marker (*cox*1 mtDNA), the second internal transcribed spacer unit of the ribosomal genome (ITS2 rDNA), a non-coding barcoding marker, and the phylogenetically informative large ribosomal subunit gene (28S rDNA). Specimens for molecular analyses were processed according to the protocols used by Cutmore et al. (2016) and Wee et al. (2017). The complete ITS2 region (with flanking 5.8S and 28S regions) was amplified and sequenced using the primers 3S (Morgan & Blair, 1995) and ITS2.2 (Cribb et al., 1998), the partial D1–D3 28S region using LSU5 (Littlewood, 1994), 300F (Littlewood et al., 2000), ECD2 (Littlewood et al., 1997) and 1500R (Snyder & Tkach, 2001), and the partial *cox*1 region using Dig_*cox*1Fa (Wee et al., 2017) and Dig_*cox*1R (Wee et al., 2017). Geneious® version 10.2.3 (Kearse et al., 2012) was used to assemble and edit contiguous sequences, which were trimmed and examined for intragenomic (i.e. intra-individual) nucleotide polymorphisms.

### Phylogenetic analyses

ITS2 sequences generated during this study were aligned with those available for species of *Siphoderina* on GenBank using MUSCLE implemented in MEGA 11 (Tamura et al., 2021), with UPGMA clustering for iterations 1 and 2. The 28S sequences generated during this study were aligned with representative sequences of related cryptogonimids available on GenBank, including comparable representative data for all sequenced species of *Siphoderina* (9 spp.), *Caulanus* (1 spp.), *Beluesca* (2 spp.), *Latuterus* (2 spp.) and *Varialvus* (3 spp.). Data for *Metadena lutiani* (Yamaguti, 1942) Miller & Cribb, 2008b, the only sequenced representative of that genus most problematically implicated with *Siphoderina*, were not included, because these data were shown to resolve relatively more distantly in the recent analyses of Miller et al. (2018). 28S data were aligned using MUSCLE v.3.7 (Edgar, 2004) run on the CIPRES portal (Miller, M., Pfeiler & Schwartz, 2010), with ClustalW sequence weighting and UPGMA clustering for iterations 1 and 2. The 28S alignment was trimmed to 858 bp; ambiguously aligned regions were few and not masked or removed. Pairwise differences for both ITS2 and 28S datasets were estimated in MEGA 11 using the following conditions: “Variance Estimation Method = None”, “Model/Method = No. of differences” and “Substitutions to Include = d: Transitions + Transversions” and “Gaps/Missing Data Treatment = Pairwise deletion”.

Phylogenetic affinities of the new material were assessed *via* maximum likelihood and Bayesian inference analyses of partial 28S rDNA sequence data. Both maximum likelihood and Bayesian inference analyses were performed *via* the CIPRES portal, using implementations of RAxML v.8.2.12 (Stamatakis, 2014) and MrBayes v.3.2.7a (Ronquist et al., 2012), respectively. The best nucleotide substitution model was estimated using jModelTest version v2.1.10 (Darriba et al., 2012); the Akaike Information Criterion (AIC) predicted the TVM+I+Γ model as the best estimator and Bayesian Information Criterion (BIC) the TPM3uf+I+Γ model; Bayesian inference and maximum likelihood analyses were conducted using the closest approximation to these models. Nodal support in the maximum likelihood analysis was estimated by performing 1,000 bootstrap pseudoreplicates. The Bayesian inference analysis was run over 10,000,000 generations (ngen = 10,000,000) and four simultaneous Markov chain Monte Carlo simulations (nchains = 4) sampled every 1,000 iterations, with the first 2,500 samples discarded as “burn-in”; the average standard deviation of split frequencies reached < 0.005. Two species of *Neometadena* Hafeezullah & Siddiqi, 1970 were included as the outgroup based on family-wide phylogenetic analyses of the Cryptogonimidae (Miller & Cribb, 2008a; Miller et al., 2018)

### Data accessibility

Raw morphometric data, the partial 28S alignment used for phylogenetic analyses, and both the partial 5.8S-ITS2-partial 28S and the partial 28S alignments used to generate pairwise distance matrices are publicly and freely available at https://data.mendeley.com/datasets/k9fg32fb3s.1.

## Results

### Molecular and phylogenetic results

**Fig. 1:**
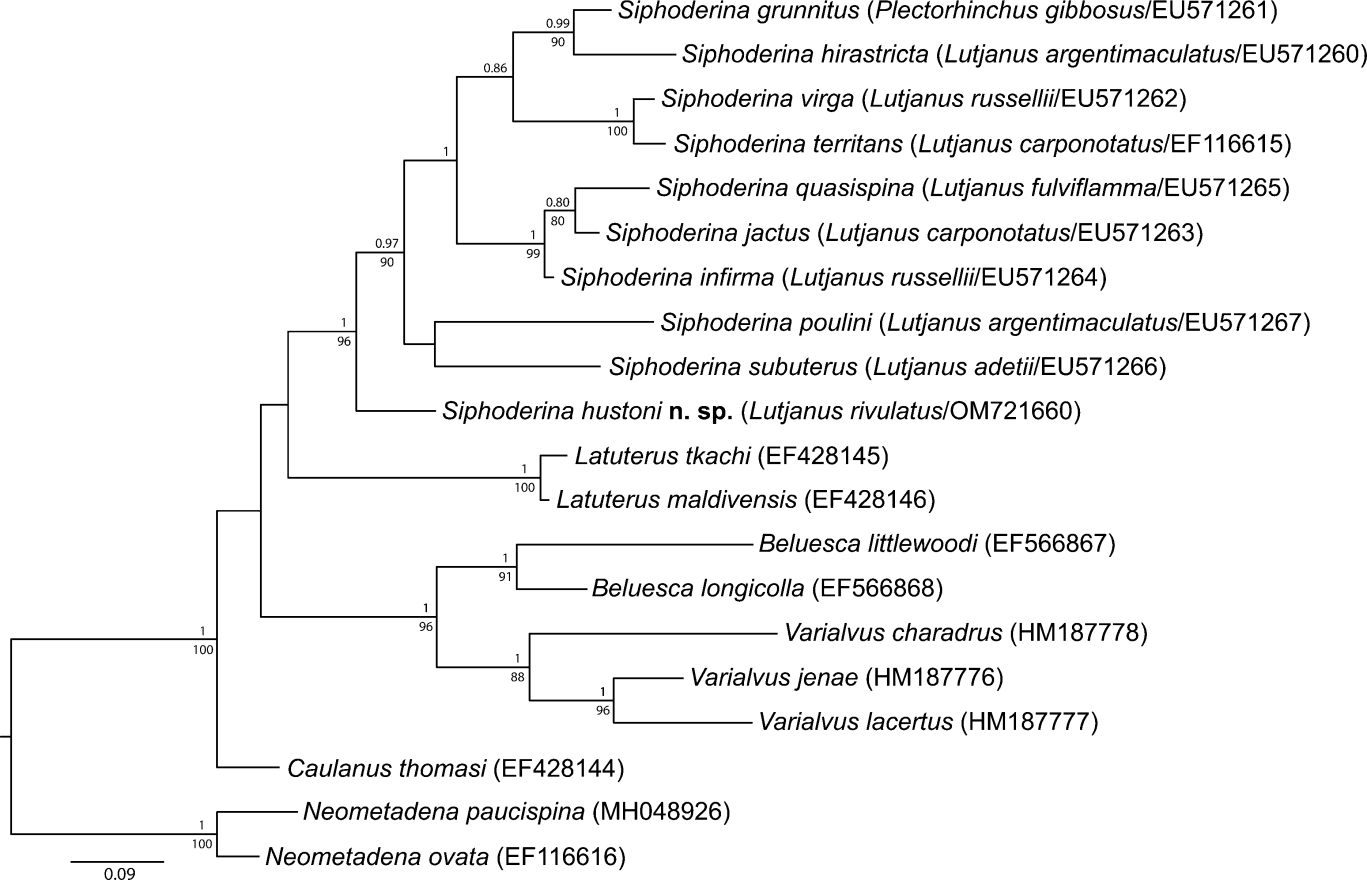
Relationships of species of *Siphoderina* Manter, 1934 and species belonging to related cryptogonimid taxa, based on Bayesian inference and maximum likelihood analyses of the 28S rDNA alignment. Posterior probabilities from the Bayesian inference analysis are shown above the nodes, with corresponding bootstrap support values from the maximum likelihood analysis below the line. Nodal support less than 0.85/85 omitted. The scale-bar indicates the expected number of substitutions per site. References for sequence data: Miller & Cribb (2007a;b;c; 2008a), Miller et al. (2010a;b; 2018).

Maximum likelihood and Bayesian inference analyses of the partial 28S rDNA alignment produced phylograms with consistent topologies (Fig. 1). Sequence data generated from the new material have greatest affinity to those from species recognised in *Siphoderina*, although the new genotype resolved basal relative to all nine species represented by genetic data. Pairwise differences calculated from both the partial 5.8S-ITS2-partial 28S and the partial 28S rDNA alignments suggest that the new genotype is similarly distinct from recognised species of *Siphoderina* as those species are from one-another (Table 1).

**Table 1:** Total pairwise ITS2 and 28S differences between species of *Siphoderina*, with number of differences in the ITS2 alignment below the diagonal and number of differences in the 28S alignment above.

	1	2	3	4	5	6	7	8	9	10
1. *S*. *grunnitus* (EU571257/EU571261)	–	14	23	25	35	26	29	22	23	28
2. *S*. *hirastricta* (EU571255/EU571260)	15	–	29	31	41	32	33	30	29	34
3. *S*. *infirma* (EU571256/EU571264)	21	19	–	5	32	11	30	25	24	25
4. *S*. *jactus* (EU571253/EU571263)	21	20	11	–	34	10	31	27	28	30
5. *S*. *poulini* (EU571254/EU571267)	20	19	26	22	–	36	37	42	39	36
6. *S*. *quasispina* (EU571259/EU571265)	19	21	15	10	20	–	31	30	29	31
7. *S*. *subuterus* (EU571252/EU571266)	33	31	31	31	31	33	–	37	34	31
8. *S*. *territans* (EF116632/EF116615)	19	20	24	15	24	21	33	–	5	28
9. *S*. *virga* (EU571258/EU571262)	24	22	22	19	30	26	30	11	–	27
10. *S*. *hustoni*** n. sp.** (OM721659/OM721660)	21	20	21	17	22	19	20	20	21	–

GenBank accession data provided in parentheses as ITS2/28S.

In the new phylogenetic analyses, represented species of *Latuterus* resolved sister to species of *Siphoderina* (including the new genotype). Species of *Beluesca* + *Varialvus* formed a clade sister to *Latuterus* + *Siphoderina*, and the representative species of *Caulanus* resolved basal to both these clades. This topology differs from previous analyses. In the analyses of Miller & Cribb (2008a), prior to the publication for data representative of species of *Varialvus*, *Beluesca* resolved sister to *Siphoderina*, and *Latuterus* sister to *Caulanus*. In the later analyses of Miller et al. (2018), *Beluesca* + *Varialvus* formed a clade as in the new analyses, but a clade comprising *Caulanus* + *Latutuerus* resolved sister to *Siphoderina*.

The topology of relationships between species within *Siphoderina* is similar in the new analyses to that of Miller & Cribb (2008a). The only difference is that in their analyses *S*. *subuterus* and then *S*. *poulini* resolved basal to the remaining species, whereas in the new analyses, *S*. *poulini* + *S*. *subuterus* formed a clade sister to all previously recognised species of *Siphoderina*.

Finally, we note that, in the new analyses, the represented species of *Beleusca* and *Varialvus* each formed monophyletic clades with strong support, but genetic distances between species of these two genera were similar to or even less than that between clades of species within *Siphoderina*.

### Taxonomy

**Cryptogonimidae Ward, 1917**

***Siphoderina*** **Manter, 1934**

**Type-species:** ***Siphoderina brotulae*** **Manter, 1934, by original designation**

***Siphoderina hustoni*** **n. sp.**

*Type-host*: *Lutjanus rivulatus* (Cuvier) (Perciformes: Lutjanidae), Maori snapper.

*Type-locality*: Coconut Bay (14°41′09″ S, 145°28′20″ E), Lizard Island, northern Great Barrier Reef, Queensland, Australia.

*Prevalence and intensity*: At least 29 specimens from a single fish examined.

*Material examined*: Holotype (QM G239729) and 23 paratypes (QM G239730–52) including four hologenophores (QM G239749-52), mounted ventrally.

*Representative DNA sequences*: Five replicates of partial 5.8S-ITS2-partial 28S rDNA (one submitted to GenBank, OM721659); one sequence of partial 28S rDNA (GB OM721660); two sequences of *cox*1 mtDNA, differing at two nucleotide positions (GB OM716679–80).

*ZooBank registration, LSID*: urn:lsid:zoobank.org:act:1AAC7468-4C33-45D6-8F00-A3802D7F41E6.

*Etymology*: This species is named for our colleague Dr Daniel C. Huston, CSIRO (Commonwealth Scientific and Industrial Research Organisation), Australia, for his heroics capturing the host fish.

Description

**Fig. 2:**
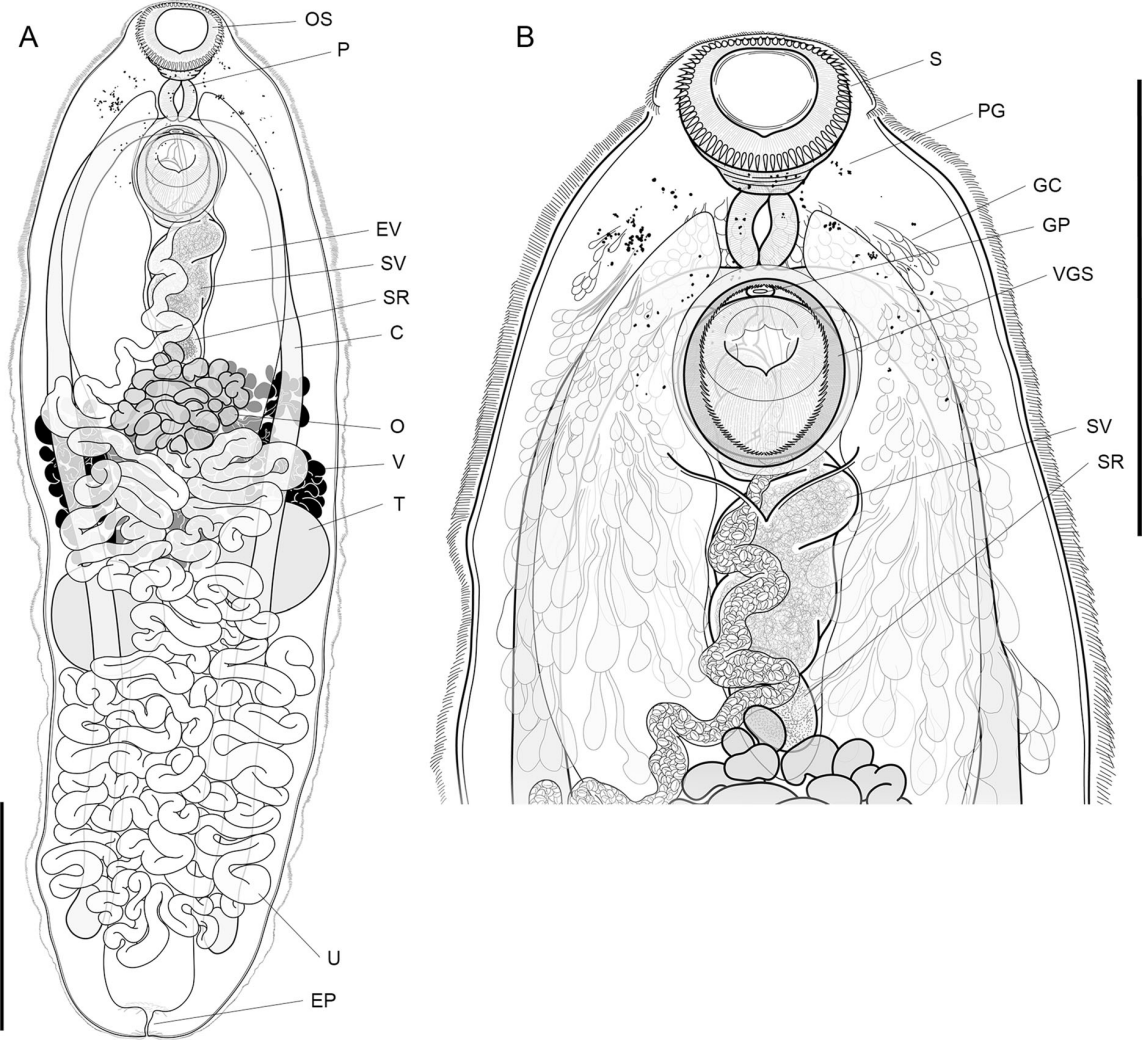
*Siphoderina hustoni* **n. sp.** holotype, ventral perspective; **A.** entire worm, **B.** anterior part. C, intestinal caeca; EV, excretory vesicle; EP, excretory pore; GC, gland cells; GP, genital pore; O, ovary; OS, oral sucker; P, pharynx; PG, remnant eye-spot pigment granules; S, oral sucker spines; SR, seminal receptacle; SV. seminal vesicle; T, testes; U, uterus; V, vitellarium; VGS, ventrogenital sac. Scale-bars: 500 µm.

[Based on 20 gravid, unflattened specimens, Fig. 2].

Body large, elongate oval, 1579–2619 (2021) × 553–758 (645), 2.76–3.6 (3.13) times longer than wide. Tegument covered with long, fine, regular spines, diminishing in posterior half of body. Forebody short, 228–325 (269) long, occupies 10–18 (14)% of body length. Remnant eyespot pigment scattered in forebody to about midlevel of ventral sucker. Gland cells, probably associated with mouth, distributed extensively dorsally and ventrally throughout forebody and midbody to level of ovary, small anteriorly, larger posteriorly. Oral sucker round, squashed funnel-shape, 125–175 (152) × 115–190 (157), 0.78–1.09 (0.97) longer than wide. Oral spines 56–73, most frequently (42%) 68 or 69, 15–21 (17) long. Ventral sucker globular, similar in size to oral sucker [0.92–1.14 (1.05) times its length and 0.76–1.13 (0.92) times its width], set within elliptical, spined cavity on ventral body surface [i.e. ventrogenital sac as per Miller & Cribb (2008b)], 139–181 (160) × 146–193 (169), 0.84–1.04 (0.95) times longer than wide. Prepharynx very short. Pharynx unspecialised, ellipsoidal, smaller than oral sucker [53–64 (58)% its length and 38–58 (49)% its width], 75–100 (88) × 59–85 (76), 1.02–1.32 (1.15) times longer than wide. Oesophagus very short. Intestinal bifurcation broad, between pharynx and ventral sucker. Intestinal caeca blind, long, mostly straight, occupy 73–81 (79)% of body length, terminate 124–302 (212) [8–15 (11)% of body length] from posterior end of body.

Testes two, round, always separate, slightly oblique, with left usually more anterior (in 65% of specimens) and right usually slightly larger; left testis 132–326 (222) × 155–299 (231); right testis 166–192 (241) × 168–294 (237); post-testicular zone 539–972 (723) long, occupies 32–41 (36)% body length. Seminal vesicle broad, medial, contorted, narrows anteriorly, 199–363 (290) × 50–139 (103), occupies 10–17 (14)% of body length. Ejaculatory duct dorsal to and about half length [32–74 (49)%] of ventral sucker, 53–106 (78) long. Genital atrium very short, simple. Genital pore medial, immediately anterior to ventral sucker, immediately postbifurcal, opens into ventrogenital sac, without gonotyl.

Ovary medial, deeply lobed, roughly rhomboid, never abuts either testis, separated from testes by 19–277 (77) [1–14 (4)% of body length], separated from ventral sucker by 189–355 (248) [10–14 (12)% of body length], situated 561–818 (677) [about one-third of body length, 29–42 (34)%] from anterior extremity, 162–265 (219) × 157–355 (245). Canalicular seminal receptacle medial, oval, smaller than and dorso-anterior to ovary and dorso-posterior to seminal vesicle, 89–233 (128) × 68–119 (85). Vitellarium composed of two branching, lateral fields of large follicles, frequently confluent, dorsal to gonads, intestinal caeca and excretory vesicle, restricted in distribution from about anterior margin of ovary to about midlevel of anterior testis and anterior margin of posterior testis; vitellarium zone 239–526 (372) long, occupies 13–22 (18)% of body length. Uterus extensive, from level of ovary to just beyond termination of intestinal caeca, ventral to testes, vitellarium, excretory vesicle and intestinal caeca, extends laterally beyond caeca near to lateral body margins but partially constrained by each testis, composed of numerous, mostly transverse coils with descending coils sinistral and ascending coils dextral; coils present between ovary and testes; final ascending coil passes dextral to ovary then medial and ventral to seminal vesicle, without discernible metraterm. Eggs small, darkly tanned, dense and numerous throughout uterus, 18–21 (19) × 9–11 (10).

Excretory vesicle Y-shaped, bifurcates dorsal to ovary; arms voluminous, pass ventral to intestinal caeca, reach to level of pharynx.

Differential diagnosis

*Siphoderina hustoni* is moderately distinctive among species recognised within *Siphoderina*. It is most readily distinguished by a spacious midbody devoid of uterine coils and vitelline follicles, separation of the ovary from the testes, an extremely short forebody, and oral and ventral suckers similar in size.

*Siphoderina hustoni* is larger than many other species in the genus (Table 2). However, several species attain larger sizes: *S*. *akamachi* Machida, 2009, *S*. *grandispinus* (Velasquez, 1961) Miller & Cribb, 2008a, *S*. *mexicana* (Bravo-Hollis, 1953) Miller & Cribb, 2008b, *S*. *nemipteri* Machida, 2009, *S*. *satyui* (Hafeezullah, 1975) [=*S*. *apharei* (Yamaguti, 1970) Miller & Cribb, 2008b], *S*. *ulaula* (Yamaguti, 1970) Miller & Cribb, 2008b, *S*. *xenocephali* Machida, 2009 and, by far the largest [11,200–12,500 µm long (Machida, 2009)], *S*. *onaga* (Yamaguti, 1970) Miller & Cribb, 2008b.

**Table 2:** Summary of morphometrics for the most useful features distinguishing species of *Siphoderina*. Measurements are sourced from original descriptions plus selected redescriptions for some tropical Indo-West Pacific species, denoted by double prime (′′). Proportional measurements estimated from published illustration denoted by tilde (~), an asterisk (*) denotes the few original reports we were unable to source. Percentages are relative to total body length.

Species	References	Body length	Body L/W	OSW/VSW	Spines	FB%	MB%	PT%
*S*. *hustoni* **n. sp.**	This study	1,579–2,619 (2,021)	2.8–3.6 (3.1)	0.9–1.3 (1.1)	56–73	10–18 (14)	~10–14(12)	32–41 (36)
*S*. *acanthostomus*	Yamaguti (1934)	1540	2.8	1.7	49	~31	~12	~24
′′	Yamaguti (1953)	3,000–3,150	~3.1	~1.7	?	~27	~18	~24
′′	Velasquez (1961)	1,360–2,320	~2.3	~1	32–34	~19	~28	~25
*S*. *ackerti*	Watson (1976)	705	2	1.7	33	19	~2	~36
*S*. *akamachi*	Machida (2009)	3,750–5,700	3.4–4.9	1.7–2.5	62–68	17–24	~14	19–32
*S*. *aloysiae*	Stossich (1885)	500–750	~1	~1.1	78	~34	~0	~33
*S*. *americana*	Manter (1940)	2,308–2,376	~2.1	1.5	52–57	~23	~5	~46
*S*. *asiatica*	Gu & Shen (1979)	952–1,503	~1.9	~1.6	65–74	~24	~14	~24
*S*. *brevicaecum*	Nahhas et al. (2003)	468–576	~1.1	2.6–3.8 (2.9)	80–90	~33	~10	~29
*S*. *brotulae*	Manter (1934)	2,600–2,850	~1.8	~4.6	?	~25	~8	~38
*S*. *catalae*	Durio & Manter (1969)	1,410–2,080	~2	1.2–1.6	56–58	20–25	~8	~33
*S*. *centropomi*	Siddiqi & Cable (1960)	851	2.5	1.3	64	~17	~5	~44
*S*. *echinostomus*	Oshmarin et al. (1961)	2,680	3.6	1.8	<20	33	~9	~20
*S*. *ghanensis*	Fischthal & Thomas (1968)	899	2.1	1.9	70–75	31	~7	~24
*S*. *grandispinus*	Velasquez (1961)	2,260–3,450	~7.4	~1.6	23–25	~31	~19	~8
*S*. *grunnitus*	Miller & Cribb (2008a)	540–894 (703)	1.5–1.8 (1.7)	1.5–2 (1.8)	67–87	26–36 (31)	~8	~29
*S*. *hirastricta*	Manter (1963)	1,482–1,920	~2.5	1.9–2.3	80–94	~26	~13	~29
′′	Miller & Cribb (2008a)	1,168–1,776 (1,443)	1.9–2.6 (2.3)	1–1.3 (1.2)	57–77	23–30 (25)	~10	~31
*S*. *infirma*	Miller & Cribb (2008a)	1,928–2,244 (2,076)	2.5–2.8 (2.7)	1.2–1.4 (1.3)	?	27–29 (28)	~13	~27
*S*. *jactus*	Miller & Cribb (2008a)	780–1,203 (1,000)	2.3–2.8 (2.5)	1.4–1.8 (1.6)	38–42	27–34 (30)	~8	~25
*S*. *leilae*	Nagaty (1957)	940–1240	~1.9	~1.6	?	33	~4	~26
*S*. *longitestis*	Durio & Manter (1969)	1,330–2,100	~2	2–2.2	?	33	~10	~10
*S*. *lutiani*	Wang (1991)	1,040–1,440	~2	~1.9	58–60	25	~8	~24
*S*. *macrospinus*	Caballero y C. et al. (1956)	1,926–3,154	~3.6	1.6–1.7	37–39	~21	~19	~28
*S*. *magna*	Winter (1958)	2,235–2,891	~1.9	1.2–1.4 (1.3)	34–36	~16	~10	~35
*S*. *magnivesiculum*	Gaevskaya & Aleshkina (1985)	1,500–1,840	~2.8	1.7	24–25	~22	~30	~16
*S*. *marangsi*	Machida (2009)	800–1,170	1.2–1.8	2.1–3.2	23–28	32–42	~40	14–25
*S*. *mexicana*	Bravo-Hollis (1953)	1,203–3,097	~8.1	1.2–1.3	?	~19	~16	~32
*S*. *microvata*	Tubangui (1928)	700–900	2–2.2	~2.5	?	~25	~11	~36
*S*. morosovi*	Parukhin (1976)	?	~2.8	~1.3	?	~20	~17	~23
*S*. *nemipteri*	Machida (2009)	2,080–4,000	3.4–4.9	1.05–1.2	28–35	20–27	~12	18–29
*S*. *neoamericana*	Siddiqi & Cable (1960)	640–884	1.9	1.5	46–51	~29	~5	~34
*S*. olmecus*	Miller & Cribb (2008a)	?	?	?	~40	?	?	?
*S*. *onaga*	Yamaguti (1970)	2,200–6,900	~2.9	~1.1	49–58	~26	~13	~19
′′	Machida (2009)	11,200–12,500	3.2–3.4	1.1	49	19–22		20–24
*S*. *paracatalae*	Durio & Manter (1969)	1,790–1,980	~2.1	1.9–2.3	65–93	~25	~10	~29
*S*. *poulini*	Miller & Cribb (2008a)	813–1,284 (1,123)	2.4–2.7 (2.6)	1.2–2 (1.7)	46–54	20–27 (23)	~12	~24
*S*. *provitellosa*	Durio & Manter (1969)	992–1,645	~2.3	1.7–2.1	~135	~37	~7	~33
*S*. *quasispina*	Miller & Cribb (2008a)	1,088–1,776 (1,505)	4.3–6.7 (5.3)	1.5–2 (1.8)	23–29	30–39 (36)	~14	~13
*S*. *ramadani*	Nahhas et al. (1998)	691–1408	~1.7–1.8	1.4–2.2	60	25–30	~10	~22–24
*S*. *rukyuensis*	Machida (2009)	1,570–2,550	2–2.8	1.6–2.0	19–25	23–33	~9	13–25
*S*. *satyui*	Yamaguti (1970)	1,950–3,800	~3.2	~1.5	38–44	~26	~12	~15
*S*. *sootai*	Hafeezullah (1975)	1,114–1,348	~1.4	2.6–3.9	?	~23	~7	~23
*S*. *subuterus*	Miller & Cribb (2008a)	920–1,378 (1,170)	1.8–2.2 (2.0)	1.6–2.0 (1.8)	66–80	22–28 (26))	~5	~33
*S*. *territans*	Miller & Cribb (2008a)	533–946 (706)	1.6–2.4 (2.0)	1.72–2 (1.9)	53–66	25–34 (29)	~7	~32
*S*. *testitactus*	Durio & Manter (1969)	2840	2.7	1.4	42–46	~25	~11	~34
*S*. *ulaula*	Yamaguti (1970)	2,200–4,500	~2.3	~1.5	42–58	~28	~10	~27
′′	Machida (2009)	3,400–5,250	1.9–2.8	0.9–1.8	44–57	19–25	?	30–40
*S*. *virga*	Miller & Cribb (2008a)	611–790 (694)	2.1–2.6 (2.3)	1.4–1.8 (1.5)	42–56	27–32 (29)	~9	~26
*S*. *xenocephali*	Machida (2009)	3,130–5,710	3.2–5.4	1.2–1.4	44–51	15–23	~14	31–43
*S*. *xiamenensis*	Liu (1996)	800–1,700	~1.8	~2	55	~25	~3	~27
*S*. *yamagutii*	Lamothe-Argumedo (1969)	3,381–3,703	~4.7	2.4	74–102	~52	~4	~24

*L* length, *W* width, *OS* oral sucker, *VS* ventral sucker, *FB* forebody (anterior to ventral sucker), *MB* midbody (measured posterior margin ventral sucker to anterior margin ovary), *PT* post-testicular zone.

Likewise, *S*. *hustoni* is also among the most elongate species in the genus, just a few other species are more elongate, although some extremely so (Table 2): *S*. *akamachi*, *S*. *grandispinus*, *S*. *mexicana*, *S*. *nemipteri*, *S*. *quasispina* Miller & Cribb, 2008a *S*. *xenocephali* and *S*. *yamagutii* (Lamonthe-Argumedo, 1969) Miller & Cribb, 2008b.

*Siphoderina hustoni* is most similar, in both size and shape, to the following species: *S*. *acanthostomus* (Yamaguti, 1934) Miller & Cribb, 2008b, *S*. *echinostomus* (Oshmarin, Mamaev & Parukhin, 1961) Miller & Cribb, 2008b, *S*. *infirma* Miller & Cribb, 2008a, and *S*. *magnivesiculum* (Gaevskaya & Aleshkina, 1985) Miller & Cribb, 2008a. It is similar in size to but more elongate than *S*. *brotulae*, *S*. *americana* (Manter, 1940) Miller & Cribb, 2008b, *S*. *catalae* (Durio & Manter, 1969) Miller & Cribb, 2008b, *S*. *magna* (Winter, 1958) Miller & Cribb, 2008b, *S*. *longitestis* (Durio & Manter, 1969) Miller & Cribb, 2008b, and *S*. *paracatalae* Durio & Manter, 1969. Likewise, it is similar in size to but substantially less elongate than *S*. *quasispina*, and comparably elongate to but smaller than *S*. *onaga* and *S*. *satyui*.

The proportions of the main regions of the body are also useful for distinguishing *S*. *hustoni*. It has a short forebody but substantial midbody and post-testicular region, where the forebody is defined as the region anterior to the ventral sucker, and the midbody as the region between the ventral sucker and gonads. These proportional measurements were rarely provided in original descriptions but can be estimated from published illustrations of identified specimens. Thus, the forebody in *S*. *hustoni* is seemingly the shortest of any species in the genus, occupying just 10–18 (14)% of body length, whereas in most species it ranges from about one-fifth to one-third; notably, in one species, *S*. *yamagutii*, the forebody occupies about half the total body length. The midbody of *S*. *hustoni* occupies 10–14 (12)% of total body length when measured from the posterior margin of the ventral sucker to the anterior margin of the ovary, which is greater than in many other species, but also comparable to several and exceeded by a few (Table 2); the proportionately largest midbody is apparently that in *S*. *magnivesiculum* (~30%). The post-testicular zone in *S*. *hustoni* is 32–41 (36)% of total body length, which appears to be among the most substantial for species in the genus. In most species, the post-testicular zone occupies about one-quarter to one-third total body length; species in which it is greater than one-third include *S*. *americana*, *S*. *brotulae*, *S*. *centropomi* (Siddiqi & Cable, 1960) Miller & Cribb, 2008b, *S*. *magna*, *S*. *ulaula* and *S*. *xenocephali*. The hindbody appears to measure about ~36% of body length in both *S*. *ackerti* (Watson, 1976) Miller & Cribb, 2008b and *S*. *microvata* (Tubangui, 1928) Miller & Cribb, 2008b, but these are small, stout species.

Related to the forebody and midbody proportions is the relative position of the ventral sucker. In *S*. *hustoni* it is positioned immediately posterior to the intestinal bifurcation, whereas in most species of the genus there is a short but distinct separation; in a few it is set significantly further posterior. The ventral sucker is similarly immediately postbifurcal in *S*. *aloysiae* (Stossich, 1885) Miller & Cribb, 2008b, *S*. *brevicaecum* (Nahhas et al., 2003) Miller & Cribb, 2008b, *S*. *brotulae*, *S*. *catalae*, and *S*. *neoamericana* (Siddiqi & Cable, 1960) Miller & Cribb, 2008b; uniquely, it is apparently immediately prebifurcal in *S*. *microvata*. The ventral sucker in *S*. *hustoni* is also among the largest for species in the genus when considered relative to the oral sucker, although several other species have similarly large ventral suckers: *S*. *aloysiae*, *S*. *brevicaecum*, *S*. *hirastricta*, *S*. *infirma*, *S*. *mexicana*, *S*. *nemipteri*, *S*. *onaga* and *S*. *xenocephali*.

*Siphoderina hustoni* is readily distinguishable from most species of *Siphoderina* by the exclusion of both the vitelline follicles and uterine coils anterior to the ovary (aside from the final ascending part of the uterus). In most species, the vitelline follicles span the midbody, that is, from about the level of the ovary to about the level of the ventral sucker; in some they are entirely clear of the ovary anteriorly, or extend posteriorly a little further to about the level of the testes, or beyond the ventral sucker anteriorly and sometimes even beyond the intestinal bifurcation to about the level of the pharynx. In *S*. *hustoni* the midbody region is substantial but void of vitelline follicles, they span only from about the midlevel of the most anterior testis to about the anterior margin of the ovary. This distribution can be considered similar only to that of *S*. *magna* and *S*. *subuterus*. The distribution is also somewhat similar to that of *S*. *ackerti* and *S*. *centropomi*, but the short midbody in these species means the vitelline follicles still reach near to the posterior margin of the ventral sucker. Likewise, the distribution is somewhat similar to that in *S*. *americana*, *S*. *catalae*, *S*. *neoamericana*, *S*. *macrospina*, *S*. *onaga*, and *S*. *testitactus*, although in these species the vitelline follicles appear to clearly extend a short distance beyond the ovary anteriorly, into the midbody but not reaching to the ventral sucker.

The uterine coils in *S*. *hustoni* are almost entirely restricted to posterior to the ovary; the ascending part briefly forms coils dextral to the ovary. A similar uterine distribution occurs in fourteen other species: *S*. *ackerti*, *S*. *akamachi*, *S*. *aloysiae*, *S*. *americana*, *S*. *catalae*, *S*. *centropomi*, *S*. *ghanensis*, *S*. *grandispinus*, *S*. *magna*, *S*. *microvata*, *S*. *neoamericana*, *S*. *olmecus*, *S*. *subuterus*, and *S*. *yamagutii*. In *S*. *leilae* and *S*. *marangsi* the uterus is distributed mostly posterior to the uterus but with substantial coils lateral to it on either side. In all other species the uterine coils extend beyond the ovary anteriorly, in many substantially so.

In *S*. *hustoni* the ovary is distinctly separate from both testes, and thus several uterine coils lie between the ovary and testes. In most other species the ovary is positioned immediately anterior to or overlaps the testes. Other species with a similar gap between the ovary and both testes include: *S*. *acanthostomus*, *S*. *akamachi*, *S*. *echinostomus*, *S*. *longitestis*, *S*. *macrospina*, *S*. *morosovi* (Parukhin, 1965) Miller & Cribb, 2008b, *S*. *onaga*, *S*. *ryukyuensis* and *S*. *satyui*. A smaller gap, allowing passage of just a few uterine coils, is present in: *S*. *americana*, *S*. *leilae*, *S*. *nemipteri*, and *S*. *ulaula*.

The oesophagus and prepharynx in *S*. *hustoni* are both extremely short, if differentiated at all. These parts of the digestive tract are not especially long in any species of the genus, but in most species one or both features are depicted as clearly longer than in *S*. *hustoni*. Other species with an extremely short oesophagus and prepharynx include: *S*. *ackerti*, *S*. *americana*, *S*. *aloysiae*, *S*. *brevicaecum*, *S*. *brotulae*, *S*. *ghanensis*, *S*. *leilae*, *S*. *magna* and *S*. *sootai*. The oral spine count for *S*. *hustoni* is intermediate and not particularly distinctive within the genus, overlapping with reported counts for multiple species.

## Discussion

*Siphoderina hustoni* is the tenth species for the genus known from Australian fishes; the other nine were reported only recently, by Miller & Cribb (2008a). All ten species are known from Queensland waters; one, *S*. *quasispina*, has also been reported from tropical Western Australian waters (Miller & Cribb, 2008a), and *S*. *hirastricta* was first reported from Fiji (Manter, 1963). Lester & Sewell (1989) reported an unidentified species of *Siphoderina* from off Heron Island, on the southern Great Barrier Reef, from the Spanish flag snapper *Lutjanus carponotatus* (Richardson); their specimens are almost certainly either *S*. *jactus* Miller & Cribb, 2008a or *S*. *territans* Miller & Cribb, 2008a, both of which are known from that host-locality combination (Miller & Cribb, 2008a), a combination which has been more intensively sampled than for any other lutjanid in Australian waters (records of T. H. Cribb).

All ten species of *Siphoderina* known from Australian waters have been sequenced with both barcode and phylogenetically informative genetic markers. However, as all ten are from typical hosts (lutjanids and haemulids) and all but one, *S*. *quasispina*, are typical in form, these molecular data contribute only limited inference for validating the breadth of the genus concept, with respect to morphological diversity, collective host-specificity, and biogeographic range. Likewise, molecular data have been published for only one nominal species of *Metadena*, the genus most problematically implicated with *Siphoderina*. In the analyses of Miller et al. (2018), data for that species, *M*. *lutiani*, did resolve distinctly separate to sequences for species of *Siphoderina*, but procurement of sequences from a greater breadth of nominal species (including morphological, host and biogeographic breadth) is needed to resolve the bounds of both genera.

The available evidence suggests that *S*. *hustoni* is likely oioxenous in its definitive host, that is, restricted to *L*. *rivulatus*. We can make this inference with some confidence because most species of *Lutjanus* inhabiting the Great Barrier Reef have been well sampled for trematodes, and all nine other species of *Siphoderina* known from Australian waters have thus far been found to exploit only one or at most two species of lutjanid or haemulid fishes (Miller & Cribb, 2008a).

Compared to its likely range of hosts, it is considerably more difficult to speculate on the potential biogeographic range of *S*. *hustoni* with any confidence. *Lutjanus rivulatus* has a broad distribution essentially spanning the entirety of the tropical Indo-West Pacific; west to East African waters, east to French Polynesia and north to the subtropical waters of southern Japan. However, it has not been well sampled for parasite fauna anywhere and so, conceivably, *S*. *hustoni* might be supported across all or most of its definitive host’s range. Most species of *Siphoderina* have been reported only from a single locality or region, although a few have reportedly broader distributions. Of these, only that of *S*. *quasispina* is supported by molecular data, and that distribution is relatively moderate, from eastern to western Australian waters. *Siphoderina acanthostomus* appears to have the broadest reported distribution within the Indo-West Pacific, originally known from Japanese waters (Yamaguti, 1934), it has since been reported from off Sulawesi (Yamaguti, 1953), the Philippines (Velasquez, 1961), the Gulf of Mannar and the Red Sea (Parukhin, 1976) and the Persian/Arabian Gulf (Kardousha, 2003). Additionally, three species, all from deeper water lutjanids, *S*. *onaga*, *S*. *satyui* and *S*. *ulaula*, have each been reported from both Hawaii (Yamaguti, 1970) and Japan (Machida, 2009; Yamaguti, 1970); *S*. *satyui* and *S*. *ulaula* have also been reported from off China (Gu & Shen, 1983; Shen, 1990) and *S*. *satyui* also from the Philippines (Velasquez, 1961). Broad tropical Indo-West Pacific distributions for several trematode species have recently been demonstrated using molecular data (Bray et al., 2021; Cutmore et al., 2021; Huston et al., 2021), including for two cryptogonimids, *Caulanus thomasi* Miller & Cribb, 2007c and *Varialvus charadrus* Miller et al., 2010b, for which sequence data were generated from specimens collected off the Great Barrier Reef and the Maldives, and also New Caledonia for the latter (Miller & Cribb, 2007c; Miller et al., 2010b).

Finally, more species of *Siphoderina* no doubt remain to be found in Australian waters. We expect them to be found mostly in those lutjanid fishes not yet well examined there. Such fishes mostly comprise species which typically reside in slightly deeper water between reefs or on outer reef slopes. Several of these fishes are regularly caught by commercial and recreational fishers; thus, procuring fishes from these sources in Australia presents a relatively easy opportunity for discovery of novel trematode biodiversity.
